# 1,6,6-Trimethyl-1*H*-chromeno[6,7-*d*]thia­zol-2(6*H*)-one

**DOI:** 10.1107/S1600536808010623

**Published:** 2008-04-23

**Authors:** Jian Tang, Yang Wang, Bei-Na Zhang, Peng Xia

**Affiliations:** a Department of Medicinal Chemistry, School of Pharmacy, Fudan University, Shanghai 200032, People’s Republic of China

## Abstract

The title compound, C_13_H_13_NO_2_S, was prepared by a thermocyclization reaction from 3-methyl-6-(2-methyl­but-3-yn-2-yl­oxy)benzo[*d*]thia­zol-2(3*H*)-one. In the crystal structure, the methyl­thia­zole unit is planar, while the pyran ring assumes a screw-boat conformation. Intra­molecular C—H⋯O hydrogen bonding helps to stabilize the molecular structure.

## Related literature

For general background, see: Gunatilaka *et al.* (1994 ▶); Ucar *et al.* (1998 ▶). For details of the synthesis, see: Delhomel *et al.* (2001 ▶).
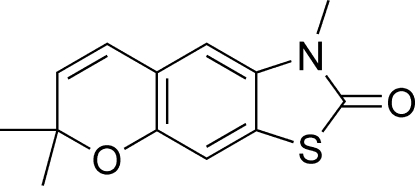


## Experimental

### 

#### Crystal data

C_13_H_13_NO_2_S
*M* *_r_* = 247.30
Triclinic, 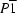

*a* = 7.376 (2) Å
*b* = 8.395 (2) Å
*c* = 10.536 (2) Å
α = 106.13 (2)°
β = 98.16 (2)°
γ = 94.08 (2)°
*V* = 616.2 (3) Å^3^
*Z* = 2
Mo *K*α radiation
μ = 0.25 mm^−1^
*T* = 298 (2) K
0.20 × 0.20 × 0.20 mm

#### Data collection

Enraf–Nonius CAD-4 diffractometer
Absorption correction: none
2765 measured reflections
2207 independent reflections
1387 reflections with *I* > 2σ(*I*)
*R*_int_ = 0.023
3 standard reflections frequency: 60 min intensity decay: 0.5%

#### Refinement

*R*[*F* ^2^ > 2σ(*F* ^2^)] = 0.076
*wR*(*F* ^2^) = 0.216
*S* = 1.05
2207 reflections
157 parameters
H-atom parameters constrained
Δρ_max_ = 0.91 e Å^−3^
Δρ_min_ = −0.68 e Å^−3^

### 

Data collection: *CAD-4 Software* (Enraf–Nonius, 1984 ▶); cell refinement: *CAD-4 Software*; data reduction: *CAD-4 Software*; program(s) used to solve structure: *SHELXTL* (Sheldrick, 2008 ▶); program(s) used to refine structure: *SHELXTL*; molecular graphics: *SHELXTL*; software used to prepare material for publication: *SHELXTL*.

## Supplementary Material

Crystal structure: contains datablocks I, global. DOI: 10.1107/S1600536808010623/xu2407sup1.cif

Structure factors: contains datablocks I. DOI: 10.1107/S1600536808010623/xu2407Isup2.hkl

Additional supplementary materials:  crystallographic information; 3D view; checkCIF report

## Figures and Tables

**Table 1:** Hydrogen-bond geometry (Å, °)

*D*—H⋯*A*	*D*—H	H⋯*A*	*D*⋯*A*	*D*—H⋯*A*
C7—H7⋯O1^i^	0.93	2.56	3.331 (5)	140

Symmetry code: (i) 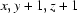.

**Fig. 1.**
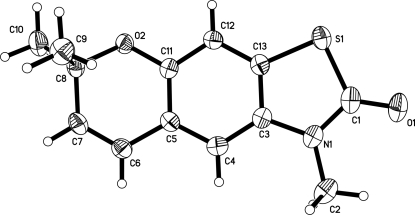
The molecular structure of (I), with atom labels and 30% probability displacement ellipsoids for non-H atoms.

**Fig. 2.**
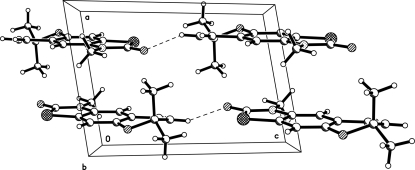
The packing of (I), viewed down the c axis, showing one demensional supra-molecular chain connected by C—H···Oi hydrogen bonding [symmetry code: (i) = x, y + 1, z + 1). H atoms not involved in hydrogen bonding have been omitted.

## supplementary crystallographic information

### Comment

2,2-Dimethyl-2*H*-benzopyran fused thiazolone is a novel potential bioactive core (Gunatilaka *et al. *1994; Ucar *et al. *1998). As part of our research program on new antitumor and antiviral agents based on bioisosterism, we synthesized the title compound and report here its crystal structure (Fig. 1).

The compound is a three rings-fused heterocycle compound. The methyl thiazole moiety shows a planar structure. The pyran ring assumes a screw-boat conformation. The C6–C7 bond distance of 1.312 (5) Å indicates a typical C═C double bond. Intramolecular C—H···O hydrogen bonding helps to stabilize the crystal structure (Table 1 and Fig. 2).

### Experimental

The title compound was synthesized by the thermo-cyclization reaction of 3-methyl-6-(2-methylbut-3-yn-2-yloxy)benzo[*d*]thiazol-2(3*H*)-one. A mixture of 6-hydroxy-3-methyl-2(3*H*)-benzothiazolone (508 mg, 2.6 mmol) (Delhomel *et al.* 2001), 3-methyl-3-chloro-but-1-yne (320 mg, 3.12 mmol) and K_2_CO_3_ (1.43 g, 10.4 mmol) was stirred in acetone (30 ml) for 5 h under reflux condition, then filtered and removed the solvent. To the residue was added N,N-diethylaniline (5 ml) and further refluxed for 2 h. The resulting solution was poured to ice water (100 ml) and extracted with acetyl acetate, and the organic layer was dried over Na_2_SO_4_, filtered, and concentrated under reduced pressure. The residue was isolated by chromatography on silica gel column with petroleum ether/EtOAc (18/1) as eluent to afford the pure compound. The solid was collected and recrystallized from acetyl acetate to give colorless crystals which were available for the single-crystal X-ray diffraction analysis. Yield: 33.5%.

### Refinement

All H atoms were positioned geometrically and refined using a riding model with C—H = 0.93 Å for aromatic H atoms and 0.96 Å for methyl H atoms, and refined in riding mode with U_iso_(H) =1.2U_eq_(C) for aromatic H atoms and U_iso_(H) = 1.5U_eq_(C) for methyl H atoms.

### Crystal data

**Table:**

C_13_H_13_NO_2_S	*Z* = 2
*M**_r_* = 247.30	*F*_000_ = 260
Triclinic, *P*1	*D*_x_ = 1.333 Mg m^−^^3^
Hall symbol: -P 1	Melting point = 376–378 K
*a* = 7.376 (2) Å	Mo *K*α radiation λ = 0.71073 Å
*b* = 8.395 (2) Å	Cell parameters from 25 reflections
*c* = 10.536 (2) Å	θ = 10.2–13.7º
α = 106.13 (2)º	µ = 0.25 mm^−^^1^
β = 98.16 (2)º	*T* = 298 (2) K
γ = 94.08 (2)º	Parallelepiped, colourless
*V* = 616.2 (3) Å^3^	0.20 × 0.20 × 0.20 mm

### Data collection

**Table:**

Enraf–Nonius CAD-4 diffractometer	*R*_int_ = 0.023
Radiation source: fine-focus sealed tube	θ_max_ = 25.2º
Monochromator: graphite	θ_min_ = 2.0º
*T* = 298(2) K	*h* = −1→8
ω/2θ scans	*k* = −10→10
Absorption correction: none	*l* = −12→12
2765 measured reflections	3 standard reflections
2207 independent reflections	every 60 min
1387 reflections with *I* > 2σ(*I*)	intensity decay: 0.5%

### Refinement

**Table:**

Refinement on *F*^2^	Secondary atom site location: difference Fourier map
Least-squares matrix: full	Hydrogen site location: inferred from neighbouring sites
*R*[*F*^2^ > 2σ(*F*^2^)] = 0.076	H-atom parameters constrained
*wR*(*F*^2^) = 0.216	*w* = 1/[σ^2^(*F*_o_^2^) + (0.1546*P*)^2^] where *P* = (*F*_o_^2^ + 2*F*_c_^2^)/3
*S* = 1.05	(Δ/σ)_max_ < 0.001
2207 reflections	Δρ_max_ = 0.91 e Å^−^^3^
157 parameters	Δρ_min_ = −0.68 e Å^−^^3^
Primary atom site location: structure-invariant direct methods	Extinction correction: none

### Special details

**Table:**

Experimental. ^1^H NMR (CDCl_3, _400 MHz): *δ* 6.87 (s, 1H, 9-H); 6.64 (s, 1H, 4-H); 6.35 (1*H*, d, *J* = 9.78 Hz, 8-H); 5.68 (d, 1H, *J *= 9.78 Hz, 7-H); 3.40 (s, 3H, 1-CH_3_); 1.43 (s, 6H, 6-CH_3_). MS: *m/z* (%) 247 (*M*^+^, 22.17).
Geometry. All e.s.d.'s (except the e.s.d. in the dihedral angle between two l.s. planes) are estimated using the full covariance matrix. The cell e.s.d.'s are taken into account individually in the estimation of e.s.d.'s in distances, angles and torsion angles; correlations between e.s.d.'s in cell parameters are only used when they are defined by crystal symmetry. An approximate (isotropic) treatment of cell e.s.d.'s is used for estimating e.s.d.'s involving l.s. planes.
Refinement. Refinement of *F*^2^ against ALL reflections. The weighted *R*-factor *wR* and goodness of fit *S* are based on *F*^2^, conventional *R*-factors *R* are based on *F*, with *F* set to zero for negative *F*^2^. The threshold expression of *F*^2^ > σ(*F*^2^) is used only for calculating *R*-factors(gt) *etc*. and is not relevant to the choice of reflections for refinement. *R*-factors based on *F*^2^ are statistically about twice as large as those based on *F*, and *R*- factors based on ALL data will be even larger.

### Fractional atomic coordinates and isotropic or equivalent isotropic displacement parameters (Å^2^)

**Table:**

	*x*	*y*	*z*	*U*_iso_*/*U*_eq_	
S1	0.72819 (14)	0.52182 (13)	−0.25523 (9)	0.0529 (4)	
N1	0.7791 (4)	0.3420 (4)	−0.0937 (3)	0.0438 (7)	
O1	0.8000 (4)	0.2061 (4)	−0.3119 (3)	0.0670 (9)	
O2	0.6410 (4)	0.9748 (3)	0.1700 (2)	0.0456 (7)	
C1	0.7748 (5)	0.3288 (5)	−0.2252 (4)	0.0510 (10)	
C2	0.8103 (5)	0.2021 (5)	−0.0421 (4)	0.0572 (11)	
H2A	0.8403	0.1115	−0.1114	0.086*	
H2B	0.7008	0.1671	−0.0128	0.086*	
H2C	0.9105	0.2348	0.0321	0.086*	
C3	0.7480 (4)	0.4981 (4)	−0.0139 (3)	0.0383 (8)	
C4	0.7478 (4)	0.5454 (4)	0.1217 (4)	0.0410 (8)	
H4	0.7695	0.4696	0.1703	0.049*	
C5	0.7150 (4)	0.7070 (4)	0.1869 (3)	0.0384 (8)	
C6	0.7087 (5)	0.7649 (5)	0.3289 (4)	0.0471 (9)	
H6	0.7087	0.6891	0.3786	0.056*	
C7	0.7028 (5)	0.9237 (5)	0.3877 (4)	0.0509 (10)	
H7	0.6938	0.9579	0.4782	0.061*	
C8	0.7103 (5)	1.0517 (4)	0.3131 (3)	0.0454 (9)	
C9	0.9067 (6)	1.1285 (5)	0.3310 (4)	0.0629 (12)	
H9A	0.9809	1.0437	0.2938	0.094*	
H9B	0.9534	1.1774	0.4248	0.094*	
H9C	0.9108	1.2132	0.2860	0.094*	
C10	0.5804 (6)	1.1829 (6)	0.3549 (4)	0.0661 (13)	
H10A	0.5912	1.2639	0.3068	0.099*	
H10B	0.6126	1.2374	0.4494	0.099*	
H10C	0.4558	1.1301	0.3349	0.099*	
C11	0.6830 (4)	0.8184 (4)	0.1118 (3)	0.0376 (8)	
C12	0.6816 (5)	0.7719 (4)	−0.0248 (3)	0.0403 (8)	
H12	0.6586	0.8470	−0.0739	0.048*	
C13	0.7152 (5)	0.6107 (4)	−0.0871 (3)	0.0407 (8)	

### Atomic displacement parameters (Å^2^)

**Table:**

	*U*^11^	*U*^22^	*U*^33^	*U*^12^	*U*^13^	*U*^23^
S1	0.0508 (6)	0.0578 (7)	0.0427 (6)	0.0037 (4)	0.0053 (4)	0.0046 (4)
N1	0.0257 (14)	0.0416 (17)	0.0569 (18)	0.0002 (12)	0.0024 (12)	0.0056 (14)
O1	0.0605 (19)	0.0622 (19)	0.0608 (18)	0.0140 (15)	0.0033 (14)	−0.0090 (15)
O2	0.0475 (15)	0.0445 (14)	0.0390 (13)	0.0094 (11)	−0.0031 (10)	0.0073 (11)
C1	0.0264 (18)	0.058 (2)	0.052 (2)	−0.0016 (16)	−0.0012 (15)	−0.0037 (18)
C2	0.032 (2)	0.050 (2)	0.082 (3)	0.0036 (17)	0.0020 (19)	0.012 (2)
C3	0.0212 (15)	0.0409 (19)	0.0482 (19)	−0.0024 (13)	0.0028 (13)	0.0083 (16)
C4	0.0258 (17)	0.046 (2)	0.050 (2)	−0.0022 (14)	−0.0010 (14)	0.0166 (17)
C5	0.0239 (16)	0.048 (2)	0.0385 (18)	−0.0033 (14)	−0.0017 (13)	0.0108 (15)
C6	0.042 (2)	0.054 (2)	0.045 (2)	0.0060 (16)	0.0023 (16)	0.0151 (17)
C7	0.047 (2)	0.065 (3)	0.0359 (18)	0.0069 (18)	−0.0001 (16)	0.0098 (18)
C8	0.041 (2)	0.049 (2)	0.0383 (19)	0.0072 (16)	−0.0015 (15)	0.0032 (16)
C9	0.046 (2)	0.066 (3)	0.067 (3)	−0.006 (2)	−0.005 (2)	0.013 (2)
C10	0.068 (3)	0.072 (3)	0.049 (2)	0.029 (2)	0.000 (2)	0.003 (2)
C11	0.0227 (16)	0.0403 (19)	0.0435 (18)	−0.0010 (13)	−0.0011 (13)	0.0065 (15)
C12	0.0338 (18)	0.044 (2)	0.0393 (18)	−0.0031 (14)	−0.0026 (14)	0.0121 (15)
C13	0.0298 (17)	0.044 (2)	0.0401 (18)	−0.0064 (14)	−0.0014 (13)	0.0060 (15)

### Geometric parameters (Å, °)

**Table:**

S1—C13	1.740 (4)	C5—C6	1.447 (5)
S1—C1	1.783 (4)	C6—C7	1.312 (5)
N1—C1	1.354 (5)	C6—H6	0.9300
N1—C3	1.401 (4)	C7—C8	1.500 (5)
N1—C2	1.444 (5)	C7—H7	0.9300
O1—C1	1.218 (4)	C8—C9	1.507 (5)
O2—C11	1.364 (4)	C8—C10	1.525 (5)
O2—C8	1.464 (4)	C9—H9A	0.9599
C2—H2A	0.9599	C9—H9B	0.9599
C2—H2B	0.9599	C9—H9C	0.9599
C2—H2C	0.9599	C10—H10A	0.9599
C3—C4	1.373 (5)	C10—H10B	0.9599
C3—C13	1.389 (5)	C10—H10C	0.9599
C4—C5	1.395 (5)	C11—C12	1.381 (5)
C4—H4	0.9300	C12—C13	1.388 (5)
C5—C11	1.395 (5)	C12—H12	0.9300
			
C13—S1—C1	91.10 (17)	C8—C7—H7	119.1
C1—N1—C3	115.4 (3)	O2—C8—C7	110.5 (3)
C1—N1—C2	121.4 (3)	O2—C8—C9	109.2 (3)
C3—N1—C2	123.2 (3)	C7—C8—C9	109.5 (3)
C11—O2—C8	118.1 (3)	O2—C8—C10	103.6 (3)
O1—C1—N1	126.6 (4)	C7—C8—C10	111.9 (3)
O1—C1—S1	123.7 (3)	C9—C8—C10	112.1 (3)
N1—C1—S1	109.8 (3)	C8—C9—H9A	109.5
N1—C2—H2A	109.5	C8—C9—H9B	109.5
N1—C2—H2B	109.5	H9A—C9—H9B	109.5
H2A—C2—H2B	109.5	C8—C9—H9C	109.5
N1—C2—H2C	109.5	H9A—C9—H9C	109.5
H2A—C2—H2C	109.5	H9B—C9—H9C	109.5
H2B—C2—H2C	109.5	C8—C10—H10A	109.5
C4—C3—C13	120.2 (3)	C8—C10—H10B	109.5
C4—C3—N1	127.2 (3)	H10A—C10—H10B	109.5
C13—C3—N1	112.6 (3)	C8—C10—H10C	109.5
C3—C4—C5	120.2 (3)	H10A—C10—H10C	109.5
C3—C4—H4	119.9	H10B—C10—H10C	109.5
C5—C4—H4	119.9	O2—C11—C12	117.5 (3)
C11—C5—C4	118.8 (3)	O2—C11—C5	120.8 (3)
C11—C5—C6	117.9 (3)	C12—C11—C5	121.6 (3)
C4—C5—C6	123.4 (3)	C11—C12—C13	118.4 (3)
C7—C6—C5	120.3 (4)	C11—C12—H12	120.8
C7—C6—H6	119.8	C13—C12—H12	120.8
C5—C6—H6	119.8	C12—C13—C3	120.8 (3)
C6—C7—C8	121.8 (3)	C12—C13—S1	128.0 (3)
C6—C7—H7	119.1	C3—C13—S1	111.2 (3)
			
C3—N1—C1—O1	179.1 (3)	C6—C7—C8—O2	25.8 (5)
C2—N1—C1—O1	−2.0 (5)	C6—C7—C8—C9	−94.5 (4)
C3—N1—C1—S1	−0.2 (3)	C6—C7—C8—C10	140.6 (4)
C2—N1—C1—S1	178.6 (2)	C8—O2—C11—C12	−155.9 (3)
C13—S1—C1—O1	−179.8 (3)	C8—O2—C11—C5	27.9 (4)
C13—S1—C1—N1	−0.4 (2)	C4—C5—C11—O2	176.6 (3)
C1—N1—C3—C4	−178.9 (3)	C6—C5—C11—O2	−2.2 (5)
C2—N1—C3—C4	2.2 (5)	C4—C5—C11—C12	0.5 (5)
C1—N1—C3—C13	1.0 (4)	C6—C5—C11—C12	−178.2 (3)
C2—N1—C3—C13	−177.9 (3)	O2—C11—C12—C13	−176.9 (3)
C13—C3—C4—C5	−0.2 (5)	C5—C11—C12—C13	−0.8 (5)
N1—C3—C4—C5	179.7 (3)	C11—C12—C13—C3	0.5 (5)
C3—C4—C5—C11	−0.1 (5)	C11—C12—C13—S1	−177.9 (3)
C3—C4—C5—C6	178.6 (3)	C4—C3—C13—C12	0.0 (5)
C11—C5—C6—C7	−10.8 (5)	N1—C3—C13—C12	−180.0 (3)
C4—C5—C6—C7	170.5 (3)	C4—C3—C13—S1	178.7 (2)
C5—C6—C7—C8	−2.6 (5)	N1—C3—C13—S1	−1.3 (3)
C11—O2—C8—C7	−37.9 (4)	C1—S1—C13—C12	179.5 (3)
C11—O2—C8—C9	82.5 (4)	C1—S1—C13—C3	1.0 (2)
C11—O2—C8—C10	−157.9 (3)		

### Hydrogen-bond geometry (Å, °)

**Table:**

*D*—H···*A*	*D*—H	H···*A*	*D*···*A*	*D*—H···*A*
C7—H7···O1^i^	0.93	2.56	3.331 (5)	140

Symmetry codes: (i) *x*, *y*+1, *z*+1.
